# The Effect of Taxifolin on Cisplatin-Induced Pulmonary Damage in Rats: A Biochemical and Histopathological Evaluation

**DOI:** 10.1155/2019/3740867

**Published:** 2019-03-12

**Authors:** Edhem Unver, Mustafa Tosun, Hasan Olmez, Mehmet Kuzucu, Ferda Keskin Cimen, Zeynep Suleyman

**Affiliations:** ^1^Department of Chest Diseases, Faculty of Medicine, Erzincan Binali Yildirim University, Erzincan, Turkey; ^2^Department of Biology, Faculty of Arts and Sciences, Erzincan Binali Yildirim University, Erzincan, Turkey; ^3^Department of Pathology, Faculty of Medicine, Erzincan Binali Yildirim University, Erzincan, Turkey; ^4^Department of Nursing, Faculty of Health Sciences, Erzincan Binali Yildirim University, Erzincan, Turkey

## Abstract

The effect of taxifolin on cisplatin-induced oxidative pulmonary damage was investigated biochemically and histopathologically in male albino Wistar rats. There were four groups, with six animals in each group: 50 mg/kg of taxifolin plus 2.5 mg/kg of cisplatin (TC) group, 2.5 mg/kg of cisplatin only (CIS) group, 50 mg/kg of taxifolin only (TG) group, and a healthy control group (HG). In terms of the experimental procedure, the animals in the TC and TG groups were first treated via oral gavage. The CIS and HG groups received distilled water as solvent, respectively. One hour later, the TC and CIS groups received cisplatin at a dose of 2.5 mg/kg (injected intraperitoneally). Taxifolin, cisplatin, and the distilled water were administered at the indicated dose and volume, using the same method daily for 14 d. At the end of this period, the animals were killed with a high dosage of thiopental anaesthesia (50 mg/kg). Blood and lung tissue samples were taken for biochemical (malondialdehyde (MDA), myeloperoxidase (MPO), total glutathione (tGSH), and 8-hydroxy-2 deoxyguanosine (8-OHdG)) analyses and histopathological examinations. The biochemical and histopathological results in the TC and HG groups were then compared with those in the CIS group. Cisplatin increased the levels of MDA, myeloperoxidase, and 8-OHdG, a marker of oxidative DNA damage, and reduced the amount of tGSH in the lung tissue. Moreover, severe alveolar damage, including oedema and extensive alveolar septal fibrosis, in addition to infiltration of polymorphic nuclear leucocytes and haemorrhagic foci, was observed in the CIS group. These histopathological findings demonstrate that taxifolin provides protection against pulmonary oxidative stress by preventing increases in oxidant parameters and decreases in antioxidants.

## 1. Introduction

Cisplatin (cis-dichlorodiammine platinum II) is a platinum compound and antineoplastic drug, with broad-spectrum activity against various solid cancers [1, 2]. The anticancer activity of cisplatin increases in accordance with dose augmentation, but increased cisplatin doses cause severe side effects [3]. The reported side effects include oxidative stress, which affects the lungs and various other tissues and organs [4]. Interstitial inflammation, fibrosis, structural pulmonary damage, and other severe complications have also been reported during cisplatin chemotherapy [5]. These adverse effects of cisplatin-induced pulmonary damage have been attributed to increased lipid peroxidation caused by free oxygen radicals and decreases in antioxidant parameters [6]. Previous research showed that cisplatin increased the amount of malondialdehyde (MDA) in animal lungs, reduced enzymatic and nonenzymatic antioxidant levels, and caused severe DNA damage [7]. In another study, the authors reported oxidative DNA damage in the lung tissue of a cisplatin-treated group [8]. In this group, the levels of total oxidants were high, whereas those of antioxidants were low [8].

Based on the literature, both oxidative stress and DNA damage appear to be important in the pathogenesis of cisplatin tissue and organ toxicity. Thus, a drug that did not induce oxidative stress and exhibited anticancer activity would reduce cisplatin-related pulmonary toxicity. Taxifolin (3,3′,4′,5,7-pentahydroxiflavanon), a flavanone found in onions, milk thistle, French maritime, and Douglas fir bark, has a known antioxidant activity [9, 10]. Furthermore, previous studies suggested that taxifolin was effective against various types of cancer [11, 12]. Thus, the literature suggests that taxifolin may reduce lung toxicity without suppressing the anticancer effects of cisplatin, possibly potentiating its anticancer activity. No previous studies have investigated the effect of taxifolin on cisplatin-induced lung toxicity. Therefore, the aim of this study was to examine the protective effect of taxifolin against cisplatin-induced oxidative pulmonary damage in rats via biochemical and histopathological analyses.

## 2. Material and Methods

### 2.1. Animals

Experimental animals were obtained from Ataturk University Medical Experimental Application and Research Centre. In total, 18 male albino Wistar rats (weight: 255–265 g) were used in the experiment. Prior to the experiment, the animals were housed in groups in a normal laboratory environment (22°C). The animal experiments were performed in accordance with the National Guidelines for the Use and Care of Laboratory Animals and were approved by the local animal ethics committee of Ataturk University (AUHADYEK), Erzurum, Turkey (decision date: 27.04.2018; no. 119).

### 2.2. Chemicals

Thiopental sodium used in the experiment was supplied by IE Ulagay (Turkey), cisplatin (Ebewa) was supplied by Liba (Turkey), and taxifolin was supplied by Evalar, (Russia).

### 2.3. Experimental Groups

The animals were divided into four groups, with six animals in each group: 50 mg/kg of taxifolin plus 2.5 mg/kg cisplatin (TC) group, 2.5 mg/kg cisplatin only (CIS) group, 50 mg/kg of taxifolin only (TG) group, and a healthy control group (HG).

### 2.4. Experimental Procedure

A 50 mg/kg dose of taxifolin was administered via oral gavage to the TC and TG groups. Distilled water as solvent was administered to the CIS and HG groups. In accordance with previous research that examined the protective effects of drugs against cisplatin toxicity [13], 1 h after the application of taxifolin and distilled water, cisplatin at a dose of 2.5 mg/kg was injected intraperitoneally in the TC and CIS groups. Taxifolin, cisplatin, and distilled water at the indicated dose and volume were administered daily for 14 d using the same method.

At the end of the 14 d period, blood samples were taken from the tail veins of the animals. The animals were then killed with a high dosage of thiopental anaesthesia (50 mg/kg), and lung tissues were removed. Malondialdehyde (MDA), myeloperoxidase (MPO), and total glutathione (tGSH) levels in the blood and lung tissue samples were measured. In addition, 8-hydroxy-2 deoxyguanosine (8-OHdG), a marker of DNA oxidative damage in the lung tissue, was measured, and histopathological examinations were carried out. The biochemical and histopathological results obtained in the TC, TG, and HG groups were compared with those in the CIS group.

### 2.5. Biochemical Analyses

#### 2.5.1. Sample Preparation

The blood samples from the tail veins were collected in separation gel Vacutainer serum tubes. The blood samples were incubated for 15 min at room temperature, and the serum was separated by centrifugation at 1,500×g for 15 min. The serum samples were stored at -80°C until used in the biochemical analysis. Prior to lung dissection, the tissue was rinsed with phosphate-buffered saline. The lung tissues were homogenized in ice-cold phosphate buffers (50 mM, pH 7.4) appropriate for the measured parameter. The tissue homogenates were centrifuged at 5,000 rpm for 20 min at 4°C, and the supernatants were extracted to analyse the tGSH, MDA, and MPO levels and protein concentrations. The protein concentration of the supernatant was measured using the Bradford method and expressed by dividing to grams of protein [14].

#### 2.5.2. MDA Levels in Serum and Tissue Samples

The measurement of MDA was based on the method of Ohkawa et al. [15], with spectrophotometric measurements of the absorbance of the pink-coloured complex formed by thiobarbituric acid and MDA. The serum/tissue homogenate sample (0.1 ml) was added to a solution containing 0.2 ml of 80 g/l of sodium dodecyl sulphate, 1.5 m of 200 g/l of acetic acid, 1.5 ml of 8 g/l 2-thiobarbiturate, and 0.3 ml of distilled water. The mixture was then incubated at 95°C for 1 h. Upon cooling, 5 ml of n-butanol : pyridine (15 : 1) was added. The mixture was vortexed for 1 min and centrifuged for 30 min at 4,000 rpm. The absorbance of the supernatant was measured at 532 nm. A standard curve was obtained using 1,1,3,3-tetramethoxypropane [15].

#### 2.5.3. MPO Activity in Serum and Tissue Samples

The method of Bradley et al. [16] was used to determine MPO activity in serum and tissue homogenates. Hydrogen peroxide (H_2_O_2_) in phosphate buffer (50 mM, pH 6) was used as substrate. Serum/tissue homogenate (20 *μ*l) was added to 280 *μ*l of assay buffer (7.0 mg of O-dianisidine HCl and 5 ml of 0.0005% H_2_O_2_ in 40 ml of phosphate buffer). The MPO activity was kinetically measured at 460 nm for 5 min [16].

#### 2.5.4. tGSH Level in Serum and Tissue Samples

The method of Sedlak and Lindsay [17] was used to measure tGSH. A cocktail solution (5.85 m of 100 mM sodium phosphate buffer, 2.8 m of 1 mM 5,5′-dithiobis(2-nitrobenzoic acid) (DTNB), 3.75 ml of 1 mM NADPH, and 80 *μ*l of 625 U/l glutathione reductase) was prepared. DTNB disulphide is chromogenic in medium. It is reduced by sulfhydryl groups and produces a yellow colour, which is measured by spectrophotometry at 412 nm. Before the measurement of tGSH, 0.1 ml of metaphosphoric acid was added to 0.1 ml of the serum/tissue homogenate and centrifuged for 2 min at 2,000 rpm for deproteinization. This cocktail solution (0.15 ml) was then added to 50 *μ*l of the supernatant. A standard curve was obtained using GSSG [17].

### 2.6. DNA Oxidation Analysis

The levels of 8-OHdG and deoxyguanosine (dG) were measured in predefined systems at various wavelengths using HPLC with HPLC-UV and HPLC-ECD electrochemical detectors. Before the HPLC analysis, the hydrolysed DNA samples were redissolved with HPLC eluent to produce a final volume of 1 ml consisting of 20 ml of the final hydrolysate. HPLC-ECD (HP, HP 1049A ECD detector, Agilent 1100 modular systems HP 1049A ECD detector, Germany) reverse-phase C18 column (250 mm × 4.6 mm × 4.0 *μ*m, Phenomenex, Torrance, CA) and a 0.05 M potassium phosphate (pH = 5.5) tampon contained acetonitrile (97 : 3, *v*/*v*), with 1 ml flow velocity per minute as the mobile phase. The dG concentration was quantified by measuring the absorbance at 245 nm, and 8-OHdG was observed with electrochemical readings (600 mV). The amounts of dG and 8-OHdG were identified using dG and 8-OHdG standards (Sigma, St. Louis, MO). 8-OHdG/10^5^ was used as a marker of DNA damage.

### 2.7. Histopathological Examination

The dissected tissues were fixed in 10% formalin solution for 24 h. 4 *μ*m thick sections were obtained from paraffin blocks following routine tissue monitoring and stained with haematoxylin and eosin (H&E). The sections were evaluated under light microscopy (Olympus BX 52; Tokyo, Japan) by a pathologist who was blinded to the treatment protocols.

### 2.8. Statistical Analysis

The data were analysed using Microsoft Excel and MedCal (Ostend, Belgium). Descriptive statistics were generated for each group. Outlier analysis was performed using Tukey's test. Differences between groups were compared by a one-way analysis of variance.

## 3. Results

### 3.1. Blood Serum and Lung Tissue MDA, MPO, and tGSH Levels

**Figure 1:**
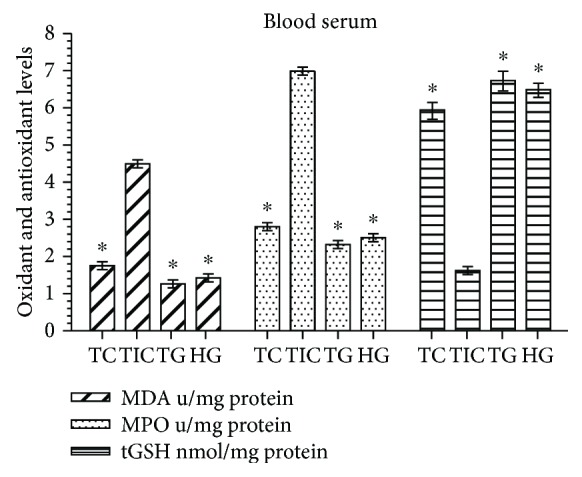
MDA, MPO, and tGSH levels in blood serum of the study groups. TC, TG, and HG groups were compared with the CIS group (*n* = 6, ^∗^*P* < 0.0001).

As shown in Figure 1, the levels of MDA and MPO in the blood serum of the cisplatin (CIS) group increased significantly as compared with those in the HG, TG, and TC groups (*P* < 0.0001). The difference between the MDA and MPO values in the HG, TG, and TC groups was statistically insignificant (*P* > 0.05). In the blood serum samples of the CIS group, the tGSH level significantly decreased as compared with that of the HG, TG, and TC groups (*P* < 0.0001). The difference between the levels of tGSH in the TG, TC, and HG groups was statistically insignificant (*P* > 0.05).

**Figure 2:**
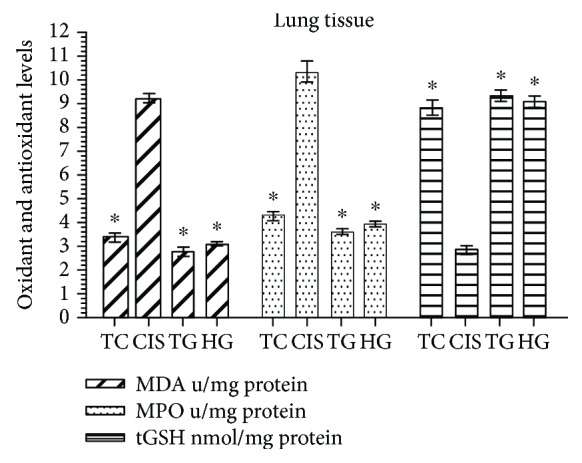
MDA, MPO and tGSH values in lung tissues of the study groups. TC, TG and HG groups were compared with the CIS group (*n* = 6, ^∗^*P* < 0.0001).

In the CIS group, the lung tissue levels of MDA and MPO significantly increased (*P* < 0.0001), whereas those of tGSH significantly decreased (*P* < 0.0001) as compared with the levels in the HG, TG, and TC groups. The lung tissue MDA, MPO, and tGSH levels were very close to those found in the HG, TG, and TC groups (Figure 2).

### 3.2. Lung Tissue Levels of 8-OHdG

**Figure 3:**
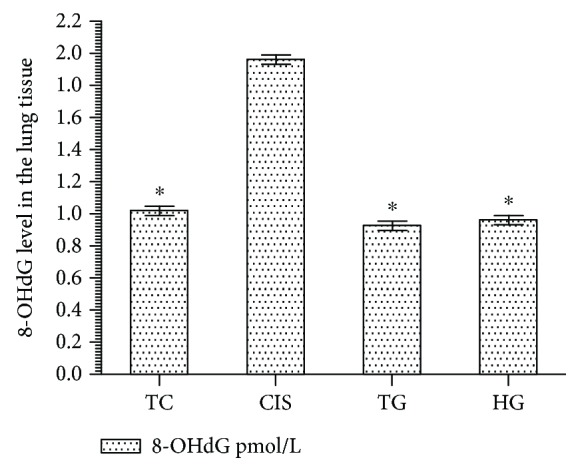
8-OHdG level in lung tissues of the study groups. TC, TG, and HG groups were compared with the CIS group (*n* = 6, ^∗^*P* < 0.0001).

In the CIS group, the lung tissue levels of 8-OHdG increased as compared with those in the HG, TG, and TC group (Figure 3). The difference between the 8-OHdG levels in the CIS, HG, TG, and TC groups was statistically significant (*P* < 0.0001). In contrast, the difference between the 8-OHdG levels in the HG, TG, and TC groups was statistically insignificant (*P* > 0.05).

### 3.3. Histopathological Findings

**Figure 4:**
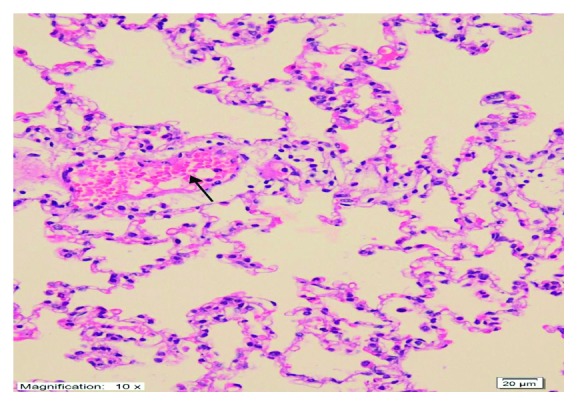
Histopathological findings (TAF) in the TC group. There were no histopathological findings, except for slight oedema and lung congestion (H&E ×200).

**Figure 5:**
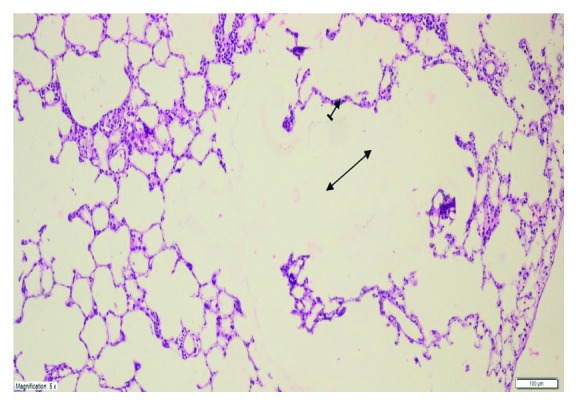
Extensive alveolar oedema (double-sided arrow) and severe alveolar damage (dashed arrow) in the lung tissue of the CIS group (H&E ×100).

**Figure 6:**
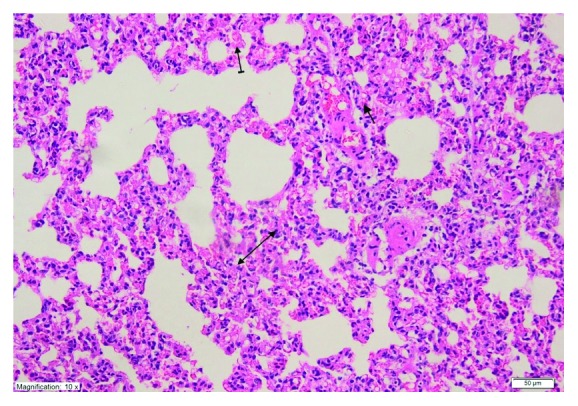
Extensive alveolar septal fibrosis (double-sided arrow), infiltration of polymorphic nuclear leucocytes (straight arrow), and haemorrhagic foci (dashed arrow) in the lung tissue of the CIS group (H&E ×200).

**Figure 7:**
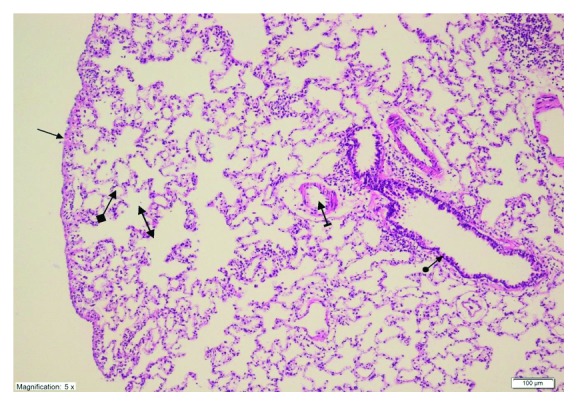
Healthy pleural mesothelium (straight arrow), healthy pulmonary arterioles (dashed arrow), normal bronchiole (round arrow) structure and alveolar structure (squared arrow), and normal histological structure of the alveolar channels (double arrow) in the lung tissue of the HG group (H&E ×100).

**Figure 8:**
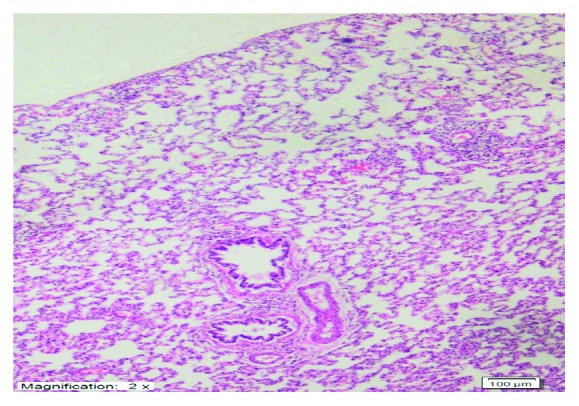
Lung tissue congestion and microscopic histopathological findings were absent in the TG group (H&E ×100).

As shown in Figure 4, no histopathological evidence of lung damage was observed in the TC group, other than slight pulmonary oedema and congestion. However, extensive alveolar oedema and severe alveolar damage were observed in the lung tissue of the CIS group (Figure 5), with extensive alveolar septal fibrosis, infiltration of polymorphic nuclear leucocytes, and haemorrhagic foci observed (Figure 6). The pleural mesothelium, pulmonary arterioles, structure of the bronchioles, and alveolar structure were all healthy in the HG (Figure 7). As observed under a microscope, the histological structure of the alveolar channels in the lung tissue of the HG was also normal (Figure 7). In the TG, lung tissue congestion and microscopic histopathological findings were absent (Figure 8).

## 4. Discussion

In this study, the effect of taxifolin on cisplatin-induced lung damage in rats was investigated biochemically and histopathologically. The results of the biochemical tests revealed significantly increased levels of MDA, MPO, and 8-OHdG and significant decreases in tGSH levels in the lung tissues of the CIS group as compared with those in the HG, TG, and TC groups.

Previous research demonstrated that various parameters, including MDA, MPO, and 8-OHdG, can be used to determine oxidative stress [18]. Free oxygen radicals give rise to oxidative stress, which oxidizes cell membrane lipids. These then form toxic products (e.g. MDA) that further increase cell damage [19]. Hypochlorous acid (HOCl), a toxic oxidant/free oxygen radical, is produced by MPO and causes lipid peroxidation [20]. MPO oxidizes H_2_O_2_ to chloride ions and leads to the formation of HOCl [19]. HOCl reacts with proteins, amino acids, lipids, and nucleic acids, causing tissue damage [21]. The release of HOCl from PNLs is upregulated in damaged tissues. Reactive oxygen species react with DNA and cause oxidative DNA damage. As a result of free radical reactions, cation exchanges in nucleic acids and chain breaks in DNA occur. If such changes cannot be repaired, DNA is mutated. 8-hydroxyguanine is considered a mutagenic form of DNA [22].

The therapeutic use of cisplatin causes oxidative stress and DNA damage in noncancerous tissues (e.g. kidney, liver, testicular, brain, and lung) [4]. According to previous research, tissue damage may be associated with a decrease in antioxidant defence mechanisms in the pathogenesis of cisplatin-induced oxidative damage [6]. In the present study, lung tissue levels of tGSH, an endogenous antioxidant molecule, significantly decreased in the CIS group as compared with those in the HG and TG groups. These findings in accordance with those of Afsar et al. [7] reported that MDA and H_2_O_2_ levels increased in the presence of cisplatin-induced lung damage, whereas the levels of GSH and other enzymatic antioxidants decreased significantly.

Taxifolin is a flavanone and a powerful antioxidant agent [23]. Flavonoids exhibit their antioxidant activity by different mechanisms, for example, by scavenging radicals [24]. These induce lipid peroxide radicals and lipid peroxidation by binding metal ions and inhibiting enzymatic reactions responsible for the formation of free radicals [24]. In previous research, taxifolin suppressed the development of oxidative stress in the lung tissue of cisplatin-treated animals. Studies also reported that taxifolin suppressed MDA production in a PC12 cell line [25] and that it provided protection against inflammatory-induced damage in the lung tissue in an experimental study by suppressing MPO activity [26]. No previous studies have investigated the effect of taxifolin on tGSH in the lung tissue or its effect on DNA oxidative damage. One study reported that GSH protected the liver tissue against ethanol-induced oxidative damage by preventing its reduction [27]. The results of the present study showed that taxifolin protected DNA from oxidative damage.

In the current study, the cisplatin group, which had high levels of oxidants and low levels of antioxidants, was characterized by extensive alveolar oedema and alveolar septal fibrosis, severe alveolar damage, infiltration of polymorphic nuclear leucocytes, and haemorrhagic foci in the lung tissue. The lung tissue of the TG group was histopathologically similar to that observed in the HG group. The presence of only slight oedema and congestion in the lung tissue of the TC group indicated that the biochemical results overlapped with the histopathological findings. Leo et al. also reported that cisplatin chemotherapy induced structural pulmonary damage associated with interstitial inflammation, fibrosis, and obliterative bronchiolitis [5].

In the clinic, cytotoxic drugs have been reported to cause pulmonary inflammation and fibrosis [28]. Antioxidants are used in the treatment of various lung diseases. Previous research reported that herbal antioxidant-based products increased the effectiveness of anticancer drugs and that they could reduce the harmful effects of these drugs [29, 30]. Impellizzeri et al. experimentally demonstrated that taxifolin inhibited lung inflammation and fibrosis by inducing anti-inflammatory activity [26].

In conclusion, cisplatin caused pulmonary oxidative stress by raising levels of oxidant and proinflammatory markers and lowering antioxidant levels. Cisplatin also caused extensive alveolar septal fibrosis. As shown by the biochemical and histopathological findings, oxidative pulmonary damage was absent in the TC group. Biochemical and histopathological manifestations of oxidative damage were not observed in the blood and lung tissues of the TG group. These findings indicate that taxifolin protected the lung tissue from the toxic effects of cisplatin. Taxifolin may be clinically effective against cisplatin-associated lung toxicity.

## Data Availability

No data were used to support this study.
